# The Correlation Structure of Local Neuronal Networks Intrinsically Results from Recurrent Dynamics

**DOI:** 10.1371/journal.pcbi.1003428

**Published:** 2014-01-16

**Authors:** Moritz Helias, Tom Tetzlaff, Markus Diesmann

**Affiliations:** 1 Institute of Neuroscience and Medicine (INM-6) and Institute for Advanced Simulation (IAS-6), Jülich Research Centre and JARA, Jülich, Germany; 2 Medical Faculty, RWTH Aachen University, Aachen, Germany; Indiana University, United States of America

## Abstract

Correlated neuronal activity is a natural consequence of network connectivity and shared inputs to pairs of neurons, but the task-dependent modulation of correlations in relation to behavior also hints at a functional role. Correlations influence the gain of postsynaptic neurons, the amount of information encoded in the population activity and decoded by readout neurons, and synaptic plasticity. Further, it affects the power and spatial reach of extracellular signals like the local-field potential. A theory of correlated neuronal activity accounting for recurrent connectivity as well as fluctuating external sources is currently lacking. In particular, it is unclear how the recently found mechanism of active decorrelation by negative feedback on the population level affects the network response to externally applied correlated stimuli. Here, we present such an extension of the theory of correlations in stochastic binary networks. We show that (1) for homogeneous external input, the structure of correlations is mainly determined by the local recurrent connectivity, (2) homogeneous external inputs provide an additive, unspecific contribution to the correlations, (3) inhibitory feedback effectively decorrelates neuronal activity, even if neurons receive identical external inputs, and (4) identical synaptic input statistics to excitatory and to inhibitory cells increases intrinsically generated fluctuations and pairwise correlations. We further demonstrate how the accuracy of mean-field predictions can be improved by self-consistently including correlations. As a byproduct, we show that the cancellation of correlations between the summed inputs to pairs of neurons does not originate from the fast tracking of external input, but from the suppression of fluctuations on the population level by the local network. This suppression is a necessary constraint, but not sufficient to determine the structure of correlations; specifically, the structure observed at finite network size differs from the prediction based on perfect tracking, even though perfect tracking implies suppression of population fluctuations.

## Author Summary

The co-occurrence of action potentials of pairs of neurons within short time intervals has been known for a long time. Such synchronous events can appear time-locked to the behavior of an animal, and also theoretical considerations argue for a functional role of synchrony. Early theoretical work tried to explain correlated activity by neurons transmitting common fluctuations due to shared inputs. This, however, overestimates correlations. Recently, the recurrent connectivity of cortical networks was shown responsible for the observed low baseline correlations. Two different explanations were given: One argues that excitatory and inhibitory population activities closely follow the external inputs to the network, so that their effects on a pair of cells mutually cancel. Another explanation relies on negative recurrent feedback to suppress fluctuations in the population activity, equivalent to small correlations. In a biological neuronal network one expects both, external inputs and recurrence, to affect correlated activity. The present work extends the theoretical framework of correlations to include both contributions and explains their qualitative differences. Moreover, the study shows that the arguments of fast tracking and recurrent feedback are not equivalent, only the latter correctly predicts the cell-type specific correlations.

## Introduction

The spatio-temporal structure and magnitude of correlations in cortical neural activity have been subject of research for a variety of reasons: the experimentally observed task-dependent modulation of correlations points at a potential functional role. In the motor cortex of behaving monkeys, for example, synchronous action potentials appear at behaviorally relevant time points [1]. The degree of synchrony is modulated by task performance, and the precise timing of synchronous events follows a change of the behavioral protocol after a phase of re-learning. In primary visual cortex, saccades (eye movements) are followed by brief periods of synchronized neural firing [2], [3]. Further, correlations and fluctuations depend on the attentive state of the animal [4], with higher correlations and slow fluctuations observed during quiet wakefulness, and faster, uncorrelated fluctuations in the active state [5]. It is still unclear whether the observed modulation of correlations is in fact employed by the brain, or whether it is merely an epiphenomenon. Theoretical studies have suggested a number of interpretations and mechanisms of how correlated firing could be exploited: Correlations in afferent spike-train ensembles may provide a gating mechanism by modulating the gain of postsynaptic cells (for a review, see [6]). Synchrony in afferent spikes (or, more generally, synchrony in spike arrival) can enhance the reliability of postsynaptic responses and, hence, may serve as a mechanism for a reliable activation and propagation of precise spatio-temporal spike patterns [7], [8], [9], [10]. Further, it has been argued that synchronous firing could be employed to combine elementary representations into larger percepts [11], [12], [7], [13], [14]. While correlated firing may constitute the substrate for some en- and decoding schemes, it can be highly disadvantageous for others: The number of response patterns which can be triggered by a given afferent spike-train ensemble becomes maximal if these spike trains are uncorrelated [15]. In addition, correlations in the ensemble impair the ability of readout neurons to decode information reliably in the presence of noise (see e.g. [16], [15], [17]). Recent studies have indeed shown that biological neural networks implement a number of mechanisms which can efficiently decorrelate neural activity, such as the nonlinearity of spike generation [18], synaptic-transmission variability and failure [19], [20], short-term synaptic depression [20], heterogeneity in network connectivity [21] and neuron properties [22] and the recurrent network dynamics [23], [24], [17]. To study the significance of experimentally observed task-dependent correlations, it is essential to provide adequate null hypotheses: Which level and structure of correlations is to be expected in the absence of any task-related stimulus or behavior? Even in the simplest network models without time varying input, correlations in the neural activity emerge as a consequence of shared input [25], [26], [27] and recurrent connectivity [24], [28], [17], [29], [30]. Irrespective of the functional aspect, the spatio-temporal structure and magnitude of correlations between spike trains or membrane potentials carry valuable information about the properties of the underlying network generating these signals [26], [28], [31], [29], [30] and could therefore help constraining models of cortical networks. Further, the quantification of spike-train correlations is a prerequisite to understand how correlation sensitive synaptic plasticity rules, such as spike-timing dependent plasticity [32], interact with the recurrent network dynamics [33]. Finally, knowledge of the expected level of correlations between synaptic inputs is crucial for the correct interpretation of extracellular signals like the local-field potential (LFP) [34].

Previous theoretical studies on correlations in local cortical networks provide analytical expressions for the magnitude [27], [24], [17] and the temporal shape [35], [36], [29], [30] of average pairwise correlations, capture the influence of the connectivity on correlations [37], [38], [28], [31], [29], [39], and connect oscillatory network states emerging from delayed negative feedback [40] to the shape of correlation functions [30]. In particular we have shown recently that negative feedback loops, abundant in cortical networks, constitute an efficient decorrelation mechanism and therefore allow neurons to fire nearly independently despite substantial shared presynaptic input [17] (see also [37], [24], [41]). We further pointed out that in networks of excitatory (E) and inhibitory (I) neurons, the correlations between neurons of different cell type (EE, EI, II) differ in both magnitude and temporal shape, even if excitatory and inhibitory neurons have identical properties and input statistics [17], [30]. It remains unclear, however, how this cell-type specificity of correlations is affected by the connectivity of the network.

The majority of previous theoretical studies on cortical circuits is restricted to local networks driven by external sources representing thalamo-cortical or cortico-cortical inputs (e.g. [42], [43], [44]). Most of these studies emphasize the role of the local network connectivity (e.g. [45]). Despite the fact that inputs from remote (external) areas constitute a substantial fraction of all excitatory inputs (about 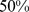 [7], see also [46], [47]), their spatio-temporal structure is often abstracted by assuming that neurons in the local network are independently driven by external sources. A priori, this assumption can hardly be justified: neurons belonging to the local cortical network receive, at least to some extent, inputs from identical or overlapping remote areas, for example due to patchy (clustered) horizontal connectivity [48], [49]. Hence, shared-input correlations are likely to play a role not only for local but also for external inputs. Coherent activation of neurons in remote presynaptic areas constitutes another source of correlated external input, in particular for sensory areas [50], [5], [51], [4]. So far, it is largely unknown how correlated external input affects the dynamics of local cortical networks and alters correlations in their neural activity.

In this article, we investigate how the magnitude and the cell-type specificity of correlations depend on i) the connectivity in local cortical networks of finite size and ii) the level of correlations in external inputs. Existing theories of correlations in cortical networks are not sufficient to address these questions as they either do not incorporate correlated external input [35], [17], [29], [28], [31] or assume infinitely large networks [24]. Lindner et al. [37] studied the responses of finite populations of spiking neurons receiving correlated external input, but described inhibitory feedback by a global compound process.

Our work builds on the existing theory of correlations in stochastic binary networks [35], a well-established model in the neuroscientific community [42], [24]. This model has the advantage of requiring for its analytical treatment elementary mathematical methods only. We employ the same network structure used in the work by Renart et al. [24] which relates the mechanism of recurrent decorrelation to the fast tracking of external signals (see [52] for a recent review). This choice enables us to reconsider the explanation of decorrelation by negative feedback [17], originally shown for networks of leaky integrate-and-fire neurons, and to compare it to the findings of Renart et al. In fact, the motivation for the choice of the model arose from the review process of [17], during which both the reviewers and the editors encouraged us to elucidate the relation of our work to the one of Renart et al. in a separate subsequent manuscript. The present work delivers this comparison.

We show here that the results presented in [17] for the leaky integrate-and-fire model are in qualitative agreement with those in networks of binary neurons. The formal relationship between spiking models and the binary neuron model is established in [53]. In particular, for weak correlations it can be shown that both models map to the Ornstein-Uhlenbeck process with one important difference: The location of the effective white noise for spiking neurons is additive in the output, while for binary neurons the effective noise is low-pass filtered, or equivalently additive on the input side of the neuron.

The remainder of the manuscript is organized as follows: In “**Methods**”, in recurrent random networks of excitatory and inhibitory cells driven by fluctuating input from an external population of finite size. We account for the fluctuations in the synaptic input to each cell, which effectively linearize the hard threshold of the neurons [54], [24]. We further include the resulting finite-size correlations into the established mean-field description [42], [54] to increase the accuracy of the theory. In “**Results**”, we first show in “**Correlations are driven by intrinsic and external fluctuations**” that correlations in recurrent networks are not only caused by the externally imposed correlated input, but also by intrinsically generated fluctuations of the local populations. We demonstrate that the external drive causes an overall shift of the correlations, but that their relative magnitude is mainly determined by the intrinsically generated fluctuations. In “**Cancellation of input correlations**”, we revisit the earlier reported phenomenon of the suppression of correlations between input currents to pairs of cells [24] and show that it is a direct consequence of the suppression of fluctuations on the population level [17]. In “**Limit of infinite network size**” we consider the strong coupling limit of the theory, where the network size goes to infinity to recover earlier results for inhomogeneous connectivity [24] and to extend these results to homogeneous connectivity. Subsequently, in “**Influence of connectivity on the correlation structure**”, we investigate in how far the reported structure of correlations is a generic feature of balanced networks and isolate parameters of the connectivity determining this structure. Finally, in “**Discussion**”, we summarize our results and their implications for the interpretation of experimental data, discuss the limitations of the theory, and provide an outlook of how the improved theory may serve as a further building block to understand processing of correlated activity.

## Methods

### Networks of binary neurons

We denote the activity of neuron 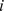 as 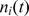. The state 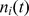 of a binary neuron is either 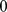 or 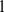, where 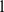 indicates activity, 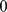 inactivity [35], [55], [24]. The state of the network of 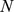 such neurons is described by a binary vector 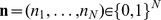. We denote the mean activity as 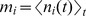, the (zero time lag) covariance of the activities of a pair 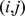 of neurons is defined as 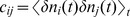, where 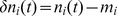 is the deviation of neuron 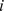
's activity from expectation and the average 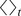 is over time and realizations of the stochastic activity.

**Figure 1:**
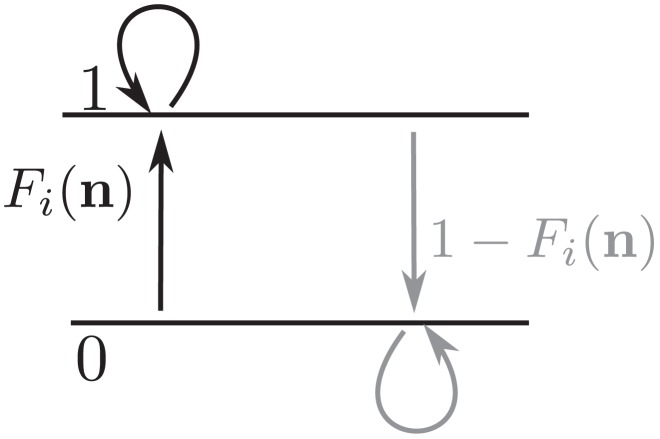
State transitions of a binary neuron. Each neuron is updated at random time points, intervals are i.i.d. exponential with mean duration 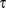, so the rate of updates per neuron 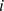 is 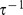. The probability of neuron 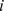 to end in the up-state (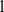) is determined by the gain function 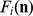 which potentially depends on the states 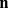 of all neurons in the network. The up-transitions are indicated by black arrows. The probability for the down state (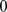) is given by the complementary probability 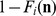, indicated by gray arrows.

The neuron model shows stochastic transitions (at random points in time) between the two states 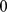 and 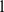 controlled by transition probabilities, as illustrated in Figure 1. Using asynchronous update [56], in each infinitesimal interval 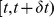 each neuron in the network has the probability 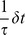 to be chosen for update [57], where 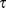 is the time constant of the neuronal dynamics. An equivalent implementation draws the time points of update independently for all neurons. For a particular neuron, the sequence of update points has exponentially distributed intervals with mean duration 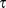, i.e. update times form a Poisson process with rate 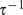. We employ the latter implementation in the globally time-driven [58] spiking simulator NEST [59], and use a discrete time resolution 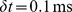 for the intervals. The stochastic update constitutes a source of noise in the system. Given the 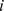-th neuron is selected for update, the probability to end in the up-state (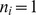) is determined by the gain function 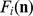 which possibly depends on the activity 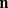 of all other neurons. The probability to end in the down state (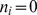) is 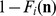. This model has been considered earlier [60], [35], [55], and here we follow the notation introduced in the latter work.

**Figure 2:**
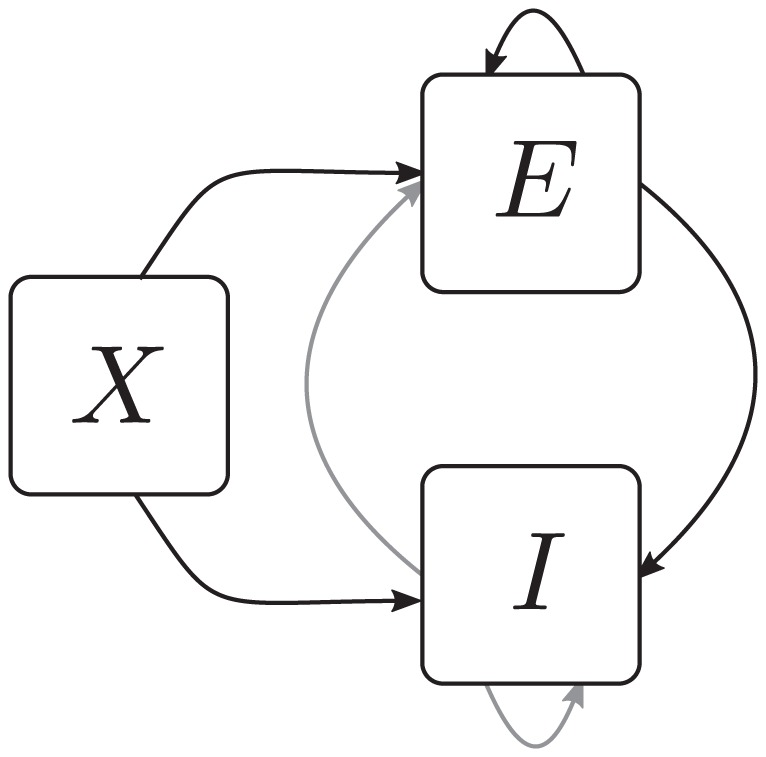
Recurrent local network of two populations of excitatory (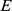) and inhibitory (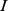) neurons driven by a common external population (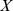). The external population 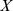 delivers stochastic activity to the local network. The local network is a recurrent Erdös-Rényi random network with homogeneous synaptic weights 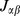 coupling neurons in population 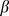 to neurons in population 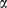, for 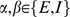 and same parameters for all neurons. There are 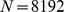 neurons in both the excitatory and the inhibitory population. The connection probability is 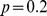, and each neuron in population 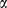 receives the same number 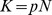 of excitatory and inhibitory synapses. The size 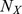 of the external population determines the amount of shared input received by each pair of cells in the local network. The neurons are modeled as binary units with a hard threshold 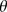.

The stochastic system is completely characterized by the joint probability distribution 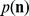 in all 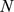 binary variables 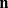. An example is the recurrent random network considered here (Figure 2). Knowing the joint probability distribution, arbitrary moments can be calculated, among them pairwise correlations. Here we are only concerned with the stationary state of the network. A stationary solution of 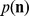 implies that for each state a balance condition holds, so that the incoming and outgoing probability fluxes sum up to zero. The occupation probability of the state is then constant. We denote as 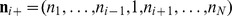 the state, where the 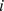-th neuron is active (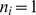), and 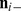 where neuron 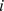 is inactive (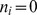). Since in each infinitesimal time interval at most one neuron can change state, for each given state 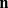 there are 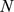 possible transitions (each corresponding to one of the 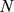 neurons changing state). The sum of the probability fluxes into the state and out of the state must compensate to zero [61], so
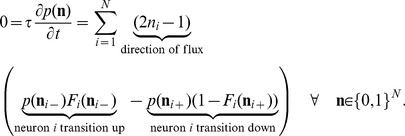
(1)

From this equation we derive expressions for the first 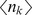 and second moments 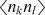 by multiplying with 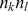 and summing over all possible states 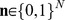, which leads to
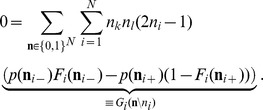
Note that the term denoted 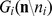 does not depend on the state of neuron 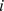. We use the notation 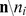 for the state of the network excluding neuron 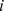, i.e. 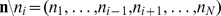. Separating the terms in the sum over 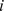 into those with 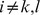 and the two terms with 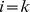 and 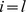, we obtain
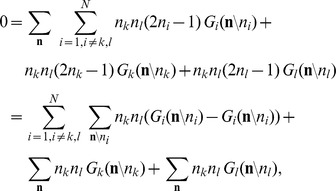
where we obtained the first term by explicitly summing over state 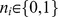 (i.e. using 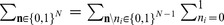 and evaluating the sum 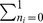). This first sum obviously vanishes. The remaining terms are of identical form with the roles of 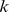 and 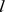 interchanged. We hence only consider the first of them and obtain the other by symmetry. The first term simplifies to
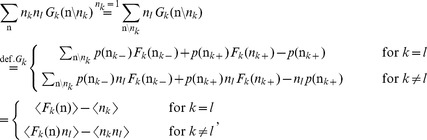
where we denote as 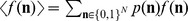 the average of a function 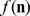 with respect to the distribution 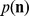. Taken together with the mirror term 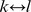, we arrive at two conditions, one for the first (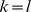, 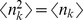) and one for the second (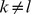) moment
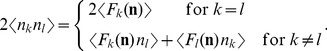
(2)Considering the covariance 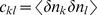 with centralized variables 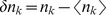, for 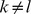 one arrives at
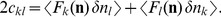
(3)This equation is identical to eq. 3.9 in [35], to eqs. 3.12 and 3.13 in [55], and to eqs. (19)–(22) in [24, supplement].

### Mean-field solution

Starting from (1) for the general case 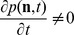, a similar calculation as the one resulting in (2) for 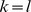 leads to
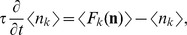
where we used 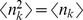, valid for binary variables. As in [24] we now assume a particular form for the gain function and for the coupling between neurons by specifying
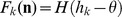

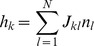

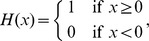
where 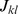 is the incoming synaptic weight from neuron 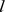 to neuron 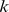, 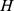 is the Heaviside function, and 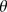 is the threshold of the activation function. For positive 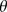 the neuron gets activated only if sufficient excitatory input is present and for negative 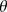 the neuron is intrinsically active even in the absence of excitatory input. We denote by 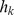 the summed synaptic input to the neuron, sometimes also called the “field”. Because 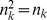, the variance 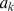 of a binary variable is 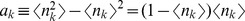. We now aim to solve (2) for the case 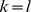, i.e. the equation 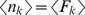. In general, the right hand side depends on the fluctuations of all neurons projecting to neuron 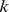. An exact solution is therefore complicated. However, for sufficiently irregular activity in the network we assume the neurons to be approximately independent. Further assume that in a network of homogeneous populations 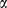 (same parameters 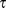, 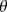 and same statistics of the incoming connections for all neurons, i.e. same number 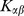 and strength 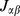 of incoming connections from neurons in a given population 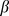) the mean activity of an individual neuron can be represented by the population mean 
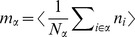
. The mean input to a neuron in population 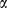 then is
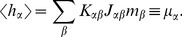
(4)We assumed in the last step identical synaptic amplitudes 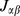 for a synapse from a neuron in population 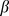 to a neuron in population 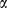. So the input to each neuron has the same mean 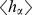. As a first approximation, if the mean activity in the network is not saturated, i.e. neither 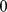 nor 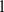, mapping this activity back by the inverse gain function to the input, 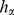 must be close to the threshold value, so
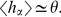
(5)This relation may be solved for 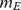 and 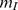 to obtain a coarse estimate of the activity in the network [42], [54]. In mean-field approximation we assume that the fluctuations of the fields of individual neurons 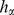 around their mean are mutually independent, so that the fluctuations 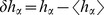 of 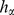 are, in turn, caused by a sum of independent random variables and hence the variances add up to the variance 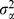 of the field
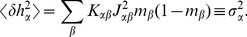
(6)As 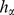 is a sum of typically thousands of synaptic inputs, it approaches a Gaussian distribution 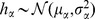 with mean 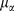 and variance 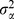. In this approximation the mean activity in the network is the solution of
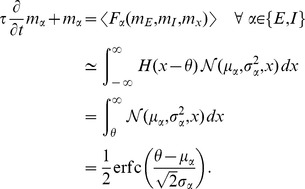
(7)This equation needs to be self-consistently solved with 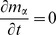 by numerical or graphical methods in order to obtain the stationary activity, because 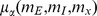 and 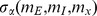 depend on 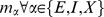 themselves. We here employ the algorithm 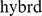 and 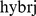 from the MINPACK package, implemented in scipy (version 0.9.0) [62] as the function 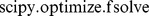.

### Linearized equation for correlations and susceptibility

In general, the term 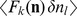 in (3) couples moments of arbitrary order, resulting in a moment hierarchy [55]. Here we only determine an approximate solution. Since the single synaptic amplitudes 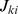 are small, we linearize the effect of a single synaptic input. We apply the linearization to the two terms of the form 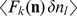 on the right hand side of (3). In the recurrent network, the activity of each neuron in the vector 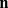 may be correlated to the activity of any other neuron 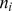. Therefore, the input 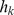 sensed by neuron 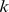 not only depends on 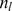 directly, but also indirectly through the correlations of 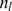 with any of the other neurons 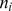 that project to neuron 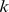. We need to take this dependence into account in the linearization. Considering the effect of one particular input 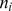 explicitly one gets
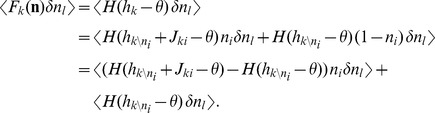
The first term 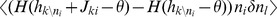 already contains two factors 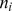 and 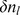, so it takes into account second order moments. Performing the expansion for the next input would yield terms corresponding to correlations of higher order, which are neglected here. This amounts to the assumption that the remaining fluctuations in 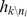 are independent of 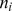 and 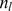, and we again approximate them by a Gaussian random variable 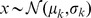 with mean 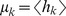 and variance 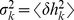, so 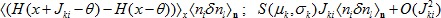. Here we used the smallness of the synaptic weight 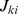 and replaced the difference by the derivative 
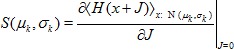
, which has the form of a susceptibility. Using the explicit expression for the Gaussian integral (7), the susceptibility is exactly
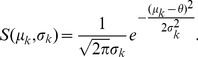
(8)The same expansion holds for the remaining inputs to cell 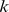. With 
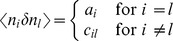
, the equation for the pairwise correlations (3) in linear approximation takes the form
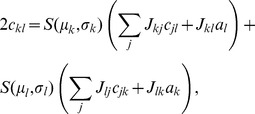
(9)corresponding to eq. (6.8) in [35] and eqs. (31)–(33) in [24, supplement]. Note, however, that the linearization used in [35] relies on the smoothness of the gain function due to additional local noise, whereas here and in [24, supplement] a Heaviside gain function is used and only the existence of noise generated by the network itself justifies the linearization. If the input to each neuron is homogeneous, i.e. 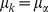 and 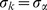 for all neurons 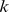 in population 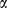, a structurally similar equation connects the correlations 
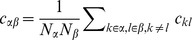
 averaged over disjoint pairs of neurons belonging to two (possibly identical) populations 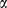, 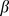 with the population averaged variances 
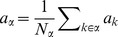

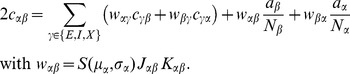
(10)In deriving the last expression, we replaced variances of individual neurons and correlations between individual pairs by their respective population averages and counted the number of connections. This equation corresponds to eqs. (9.14)–(9.16) in [35] (which lack, however, the external population 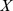, and note the typo in the first term in line 2 of eq. (9.16), which should read 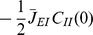) and eqs. (36) in [24, supplement]. Written in matrix form (10) takes the form (24) stated in the results sections of the present article, where we defined
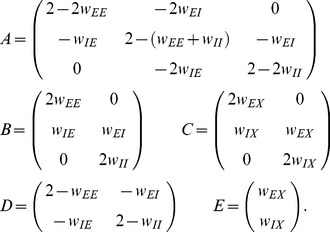
(11)The explicit solution of the system of equations in the second line of (24) is
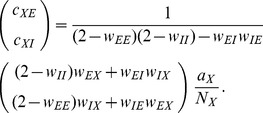
(12)

### Mean-field theory including finite-size correlations

**Figure 3:**
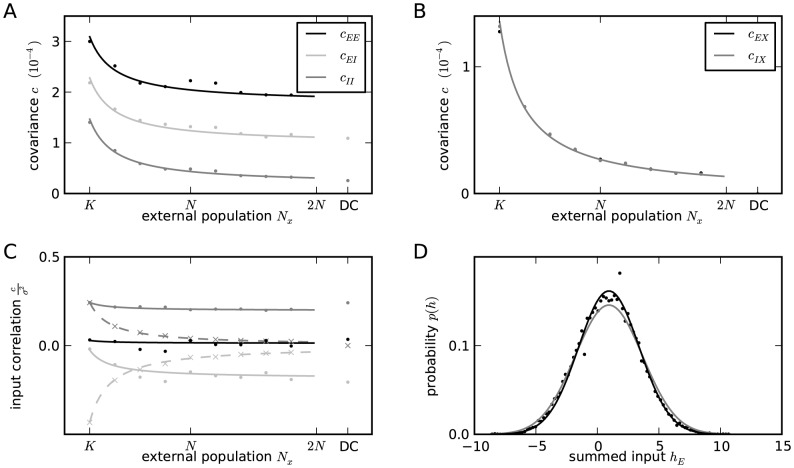
Correlations in a network of three populations as illustrated in Figure 2 in dependence of the size 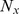 of the external population. Each neuron in population 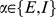 receives 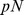 randomly drawn excitatory inputs with weight 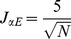, 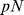 randomly drawn inhibitory inputs of weight 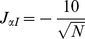 and 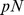 external inputs of weight 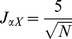 (homogeneous random network with fixed in-degree, connection probability 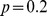). **A** Correlations averaged over pairs of neurons within the local network (22). Dots indicate results of direct simulation over 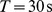 averaged over 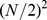 pairs of neurons. Curves show the analytical result (24). The point **“**DC**”** shows the correlation structure emerging if the drive from the external population is replaced by a constant value 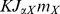, which provides the same mean input as the original external drive. **B** Correlations between neurons within the local network and the external population averaged over pairs of neurons (same labeling as in A). **C** Correlation between the inputs to a pair of cells in the network decomposed into the contributions due to shared inputs 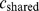 (gray, eq. 25) and due to correlations 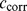 in the presynaptic activity (light gray, eq. 26). Dashed curves and St. Andrew's Crosses show the contribution due to external inputs, solid curves and dots show the contribution from local inputs. The sum of all components is shown by black dots and curve. Curves are theoretical results based on (24), (25), and (26), symbols are obtained from simulation. **D** Probability distribution of the fluctuating input 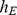 to a single neuron in the excitatory population. Dots show the histogram obtained from simulation binned over the interval 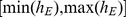 with a bin size of 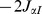. The gray curve is the prediction of a Gaussian distribution obtained from mean-field theory neglecting correlations, with mean and variance given by (4) and (6), respectively. The black curve takes correlations in the afferent signals into account and has a variance given by (13). Other parameters: simulation resolution 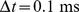, synaptic delay 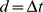, activity measurement in intervals of 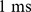. Threshold of the neurons 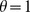, time constant of inter-update intervals 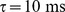. The average activity in the network is 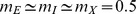.

The mean-field solution presented in “**Mean-field solution**” assumes that correlations among the neurons in the network are negligible. This assumption enters the expression (6) for the variance of the input to a neuron. Having determined the actual magnitude of the correlations in (24), we are now able to state a more accurate approximation in which we take these correlations into account, modifying the expression for the variance of the field 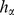

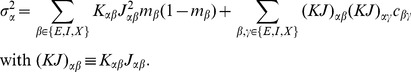
(13)This correction suggests an iterative scheme: Initially we solve the mean-field equation (7) assuming 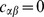 (hence 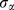 given by (6)). In each step of the iteration we then calculate the correlations by (24), compute the mean-field solution of (7) and the susceptibility 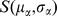 (8), taking into account the correlations (13) determined in the previous step. These steps are iterated until the solution (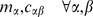) converges. We use this approach to determine the correlation structure in Figure 3, where we iterated until the solution became invariant up to a residual absolute difference of 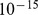. A comparison of the distribution of the total synaptic input 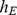 at the end of the iteration with a Gaussian distribution with parameters 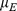 and 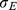 is shown in Figure 3D.

### Influence of inhomogeneity of in-degrees

In the previous sections we assumed the number of incoming connections to be the same for all neurons. Studying a random network in its original Erdös-Rényi [63] sense, the number of synaptic inputs 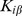 to a neuron 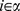 from population 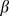 is a binomially distributed random number. As a consequence, the time-averaged activity differs among neurons. Since each neuron 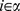 samples a random subset of inputs from a given population 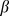, we can assume that the realization of 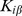 is independent of the realization of the time-averaged activity of the inputs from population 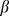. So these two contributions to the variability of the mean input 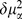 add up. The number of incoming connections to a neuron in population 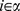 follows a binomial distribution
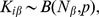
where 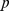 is the connection probability and 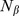 the size of the sending population. The mean value is as before 
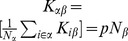
, where we denote the expectation value with respect to the realization of the connectivity as 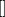. The variance of the in-degree is hence
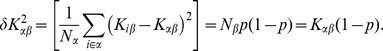
In the following we adapt the results from [54], [24] to the present notation. The contribution of the variability of the number of synapses to the variance of the mean input is 
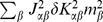
. The contribution from the distribution of the mean activities can be expressed by the variance of the mean activity defined as
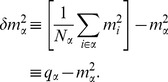
The 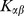 independently drawn inputs hence contribute 
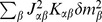
, as the variances of the 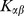 terms add up. So together we have [54, eq. 5.5–5.6]
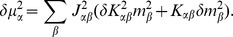
Using 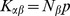 we obtain
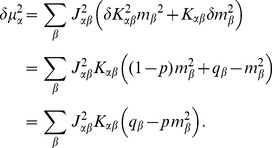
(14)The latter expression differs from [54, eq. 5.7] only in the term 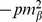 that is absent in the work of van Vreeswijk and Sompolinsky, because they assumed the number of synapses to be Poisson distributed in the limit of sparse connectivity [54, Appendix, (A.6)] (also note that their 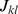 corresponds to our 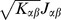). The expression (14) is identical to [24, supplement, eq. (25)].

**Figure 4:**
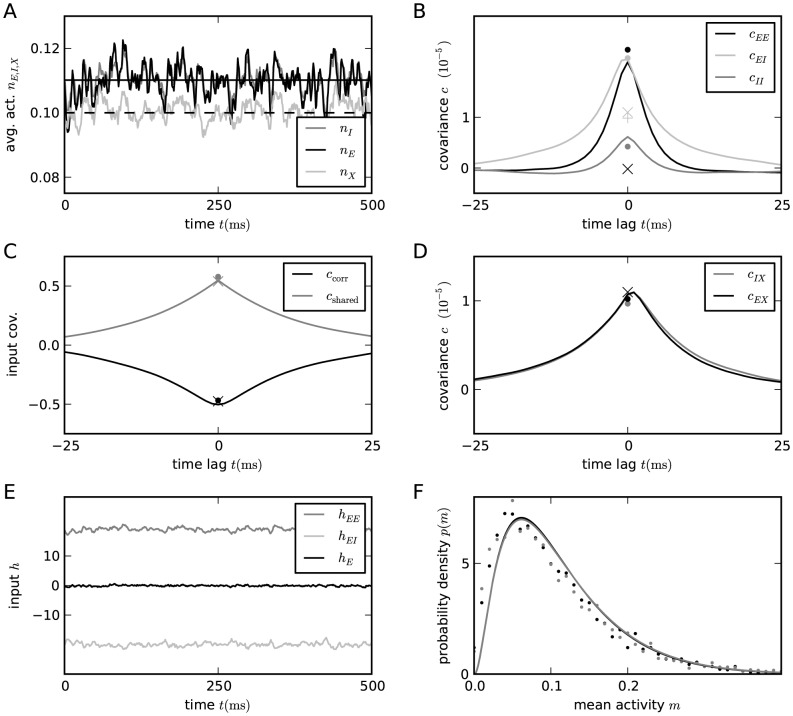
Activity in a network of 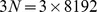 binary neurons as described in [24, their Fig. 2], with 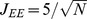, 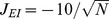, 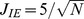, 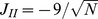, 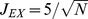, 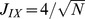. Number 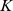 of synaptic inputs binomially distributed as 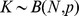, with connection probability 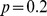. **A** Population averaged activity (black 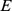, gray 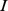, light gray 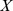). Analytical prediction (5) for the mean activities 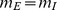 (dashed horizontal line) and numerical solution of mean field equation (7) (solid horizontal line). **B** Cross correlation between excitatory neurons (black curve), between inhibitory neurons (gray curve), and between excitatory and inhibitory neurons (light gray curve) obtained from simulation. St. Andrew's Crosses show the theoretical prediction from [24, supplement, eqs. 38,39] (prediction yields 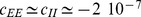, so only one cross is visible). Dots show the theoretical prediction (24). The plus symbol shows the prediction for the correlation 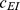 when terms proportional to 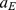 and 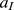 are set to zero. **C** Correlation between the input currents to a pair of excitatory neurons. Contribution due to pairwise correlations 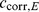 (black curve) and due to shared input 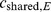 (gray curve). Symbols show the theoretical predictions based on [24] (crosses) and based on (24) (dots). **D** Similar to B, but showing the correlations between external neurons and neurons in the excitatory and inhibitory population. **E** Fluctuating input 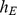 averaged over the excitatory population (black), separated into contributions from excitatory synapses 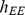 (gray) and from inhibitory synapses 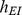 (light gray). **F** Distribution of time averaged activity obtained by direct simulation (symbols) and analytical prediction (17) using the numerically evaluated self-consistent solution for the first 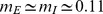 and second moments 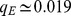, 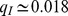 (19). Duration of simulation 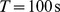, mean activity 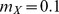, other parameters as in Figure 3.

Since the variance of a binary signal with time-averaged activity 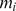 is 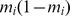, the population-averaged variance is hence
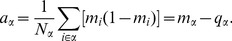
(15)So the sum of 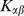 such (uncorrelated) signals contributes to the fluctuation of the input as
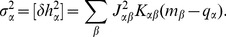
(16)The contribution due to the variability of the number of synapses 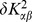 can be neglected in the limit of large networks [24]. With the time-averaged activity of a single cell with mean input 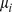 and variance 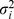 given by (7) 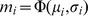 the distribution of activity in the population is
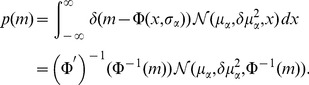
(17)The mean activity of the whole population is
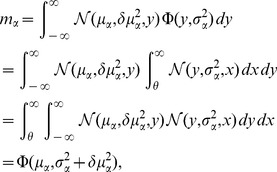
(18)because the penultimate line is a convolution of two Gaussian distributions, so the means and variances add up. The second moment of the population activity is
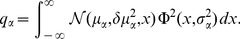
(19)These expressions are identical to [24, supplement, eqs. (26), (27)]. The system of equations (4), (14), (16), (18), and (19) can be solved self-consistently. We use the algorithm 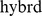 and 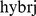 of the MINPACK package, implemented in scipy (version 0.9.0) [62] as the function 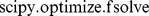. This yields the self-consistent solutions for 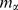 and 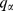 and hence the distribution of time averaged activity (17) can be obtained, shown in Figure 4F.

## Results

Our aim is to investigate the effect of recurrence and external input on the magnitude and structure of cross-correlations between the activities in a recurrent random network, as defined in **“Networks of binary neurons”**. We employ the established recurrent neuronal network model of binary neurons in the balanced regime [42]. The binary dynamics has the advantage to be more easily amendable to analytical treatment than spiking dynamics and a method to calculate the pairwise correlations exists [35]. The choice of binary dynamics moreover renders our results directly comparable to the recent findings on decorrelation in such networks [24]. Our model consists of three populations of neurons, one excitatory and one inhibitory population which together represent the local network, and an external population providing additional excitatory drive to the local network, as illustrated in Figure 2. The external population may either be conceived as representing input into the local circuit from remote areas or as representing sensory input. The external population contains 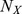 neurons, which are pairwise uncorrelated and have a stochastic activity with mean 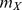. Each neuron in population 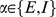 within the local network draws 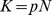 connections randomly from the finite pool of 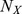 external neurons. 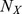 therefore determines the number of shared afferents received by each pair of cells from the external population with on average 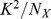 common synapses. In the extreme cases 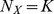 all neurons receive exactly the same input, whereas for large 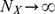 the fraction of shared external input approaches 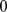. The common fluctuating input received from the finite-sized external population hence provides a signal imposing pairwise correlations, the amount of which is controlled by the parameter 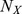.

### Correlations are driven by intrinsic and external fluctuations

To explain the correlation structure observed in a network with external inputs (Figure 2), we extend the existing theory of pairwise correlations [35] to include the effect of externally imposed correlations. The global behavior of the network can be studied with the help of the mean-field equation (7) for the population-averaged mean activity 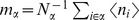

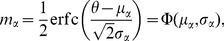
(20)where the fluctuations of the input 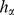 to a neuron in population 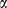 are to good approximation Gaussian with the moments
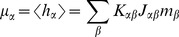
(21)
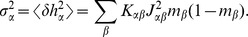
To determine the average activities in the network, the mean-field equation (20) needs to be solved self-consistently, as the right-hand side depends on the mean activities 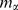 through (21), as explained in **“Mean-field theory including finite-size correlations”**. Here 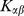 denotes the number of connections from population 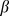 to 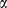, and 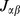 their average synaptic amplitude. Once the mean activity in the network has been found, we can determine the structure of correlations. For simplicity we focus on the zero time lag correlation, 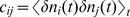, where 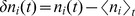 is the deflection of neuron 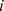
's activity from baseline and 
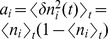
 is the variance of neuron 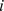
's activity. Starting from the master equation for the network of binary neurons, in **“Methods”** for completeness and consistency in notation we re-derive the self-consistent equation that connects the cross covariances 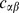 averaged over pairs of neurons from population 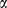 and 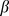 and the variances 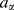 averaged over neurons from population 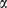

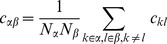
(22)
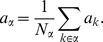
The obtained inhomogeneous system of linear equations (24) reads [35]
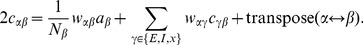
(23)Here 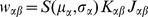 measures the effective linearized coupling strength from population 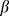 to population 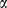. It depends on the number of connections 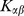 from population 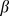 to 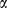, their average synaptic amplitude 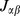 and the susceptibility 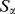 of neurons in population 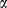. The susceptibility 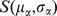 given by (8) quantifies the influence of fluctuation in the input to a neuron in population 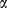 on the output. 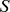 depends on the working point 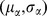 of the neurons in population 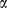. The autocorrelations 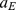, 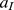 and 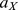 are the inhomogeneity in the system of equations, so they drive the correlations, as pointed out earlier [35]. This is in line with the linear theories [17], [30] for leaky integrate-and-fire model neurons, where cross-correlations are proportional to the auto-correlations; the system of equations (23) is identical to [35, eqs. (9.14)–(9.16)]. Note that this description holds for finite-sized networks. With the symmetry 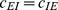, (23) can be written in matrix form as
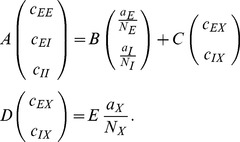
(24)The explicit forms of the matrices 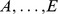 are given in (11). This system of linear equations can be solved by elementary methods. From the structure of the equations it follows, that the correlations between the external input and the activity in the network, 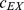 and 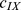, are independent of the other correlations in the network. They are solely determined by the solution of the system of equations in the second line of (24), driven by the fluctuations of the external drive 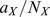. The correlations among the neurons within the network are given by the solution of the first system in (24). They are hence driven by two terms, the fluctuations of the neurons within the network proportional to 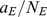 and 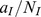 and the correlations between the external population and the neurons in the network, 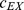 and 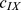.

The second line of (24) shows that all correlations depend on the size 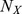 of the external population. Since the number 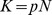 of randomly drawn afferents per neuron from this population is constant, the mean number of shared inputs to a pair of neurons is 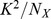. In the extreme case 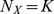 on the left of Figure 3 all neurons receive exactly identical input. If the recurrent connectivity would be absent, we would hence have perfectly correlated activity within the local network, the covariance between two neurons would be equal to their variance 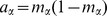, in this particular network 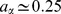. Figure 3A shows that the covariance in the recurrent network is much smaller; on the order of 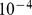. The reason is the recently reported mechanism of decorrelation [24], explained by the negative feedback in inhibition-dominated networks [17]. Increasing the size of the external population decreases the amount of shared input, as shown in Figure 3C. In the limit where the external drive is replaced by a constant value (visualized as point “
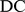
”), the external drive does consequently not contribute to correlations in the network. Figure 3A shows that the relative position of the three curves does not change with 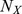. The overall offset, however, changes. This can be understood by inspecting the analytical result (24): The solution of this system of linear equations is a superposition of two contributions. One is due to the externally imposed fluctuations, proportional to 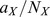, the other is due to fluctuations generated within the local network, proportional to 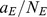 and 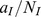. Varying the size of the external population only changes the external contribution, causing the variation in the offset, while the internal contribution, causing the splitting between the three curves, remains constant. In the extreme case 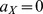 (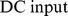), we still observe a similar structure. The slightly larger splitting is due to the reduced variance 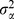 in the single neuron input, which consequently increases the susceptibility 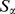 (8).

Figure 3D shows the probability distribution of the input 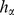 to a neuron in population 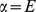. The histogram is well approximated by a Gaussian. The first two moments of this Gaussian are 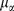 and 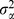 given by (21), if correlations among the afferents are neglected. This approximation deviates from the result of direct simulation. Taking the correlations among the afferents into account affects the variance in the input according to (13). The latter approximation is a better estimate of the input statistics, as shown in Figure 3D. This improved estimate can be accounted for in the solution of the mean-field equation (20), which in turn affects the correlations via the susceptibility 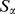. Iterating this procedure until convergence, as explained in **“Mean-field theory including finite-size correlations”**, yields the semi-analytical results presented in Figure 3.

### Cancellation of input correlations

For strongly coupled networks in the limit of large network size, previous work [24], [52] derived a balance equation for the correlations between pairs of neurons. The expressions for the correlations are approximate at finite network size and become exact for infinitely large networks. The authors show that the resulting structure of correlations amounts to a suppression of the correlations between the input currents to a pair of cells and that the population-averaged activity closely follows the fluctuations imposed by the external drive, known as fast tracking [42]. Here we revisit these three observations - the correlation structure, the input correlation, and fast tracking - from a different view point, providing an explanation based on the suppression of population rate fluctuations by negative feedback [17].

Figure 4A shows the population activities in a network of three populations for fixed numbers of neurons 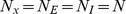 and otherwise identical parameters as in [24, their Fig. 2]. Moreover, we distributed the number of incoming connections 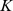 per neuron according to a binomial distribution as in the original publication. The deflections of the excitatory and the inhibitory population partly resemble those of the external drive to the network, but partly the fluctuations are independent. Our theoretical result for the correlation structure (24) is in line with this observation: the fluctuations in the network are not only driven by external input (proportional to 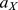), but also by the fluctuations generated within the local populations (proportional to 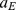 and 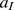), so the tracking cannot be perfect in finite-sized networks.

We now consider the fluctuations in the input averaged over all neurons 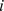 belonging to a particular population 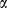, 
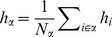
. We can decompose the input 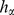 to the population 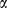 into contributions from excitatory (local and external) and from inhibitory cells, 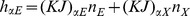 and 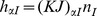, respectively, where we used the short hand 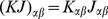. As shown in Figure 4E, the contributions of excitation and inhibition cancel each other so that the total input fluctuates close to the threshold (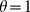) of the neurons: the network is in the balanced state [42]. Moreover, this cancellation not only holds for the mean value, but also for fast fluctuations, which are consequently reduced in the sum 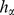 compared to the individual components 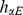 and 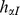 (Figure 4E).

We next show that this suppression of fluctuations directly implies a relation for the correlation 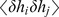 between the inputs to a pair 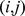 of individual neurons. There are two distinct contributions to this correlation 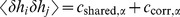, one due to common inputs shared by the pair of neurons (both neurons 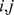 assumed to belong to population 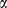)
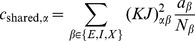
(25)and one due to the correlations between afferents
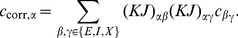
(26)Figure 4C shows these two contributions to be of opposite sign but approximately same magnitude, as already shown in [24, supplement] and in [17]. Figure 3C shows a further decomposition of the input correlation into contributions due to the external sources and due to connections from within the local network. The sum of all components is much smaller than each individual component. This cancellation is equivalent to small fluctuations in the population-averaged input 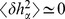, because
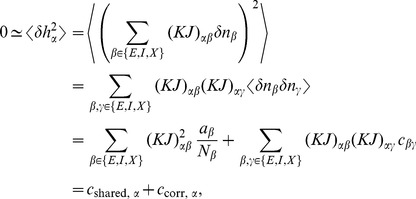
(27)where in the second step we used the general relation between the covariance 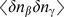 among two population averaged signals 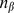 and 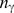, the population-averaged variance 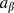, and the pairwise averaged covariances 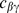, which reads [17, cf. eq. (1)]
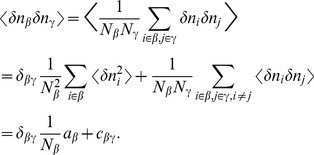
(28)We have therefore shown that the cancellation of the contribution of shared input 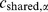 with the contribution due to the correlations among cells 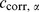 is equivalent to a suppression of the fluctuations in the population-averaged input signal to the population 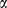.

**Figure 5:**
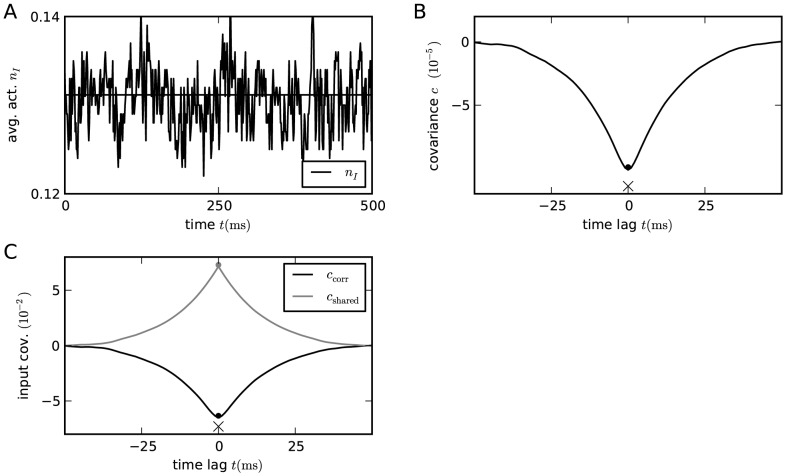
Suppression of correlations by purely inhibitory feedback in absence of external fluctuations. Activity in a network of 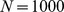 binary inhibitory neurons with synaptic amplitudes 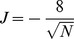. Each neuron receives 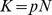 randomly drawn inputs (fixed in-degree) with 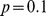. **A** Population averaged activity. Numerical solution of mean field equation (7) (solid horizontal line). **B** Cross covariance between inhibitory neurons. Theoretical result (32) shown as dot. St. Andrew's Cross indicates the leading order term 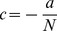. **C** Correlation between the input currents to a pair of excitatory neurons. The black curve is the contribution due to pairwise correlations 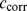, the gray curve is the contribution of shared input 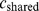. The dot symbols show the theoretical expectations (33) based on the leading order (crosses) and based on the full solution (32) (dot). Threshold of neurons 
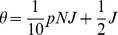
.

This suppression of fluctuations in the population-averaged input is a consequence of the overall negative feedback in these networks [17]: a fluctuation 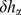 of the population averaged input 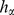 causes a response in network activity which is coupled back with a negative sign, counteracting its own cause and hence suppressing the fluctuation 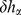. Expression (27) is an algebraic identity showing that hence also correlations between the total inputs to a pair of cells must be suppressed. Qualitatively this property can be understood by inspecting the mean-field equation (7) for the population-averaged activities, where we linearized the gain function 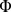 around the stationary mean-field solution to obtain
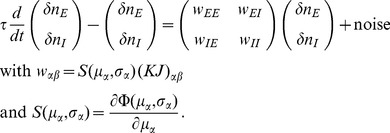
(29)Here the noise term qualitatively describes the fluctuations caused by the stochastic update process and the external drive (see [53] for the appropriate treatment of the noise). After transformation into the coordinate system of eigenvectors 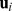 (with eigenvalue 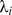) of the effective connectivity matrix 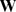, each component fulfills the differential equation
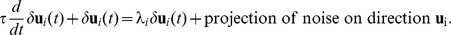
For stability the eigenvalues must satisfy 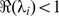. In the example of the 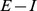 network shown in Figure 4 we have the two eigenvalues
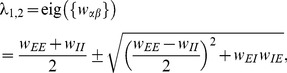
(30)which in the case of identical susceptibility 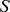 for all populations can be expressed in terms of the synaptic weights
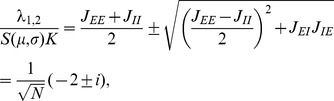
(31)where in the second line we inserted the numerical values of Figure 4. The fluctuations 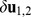 are hence suppressed so the contributions 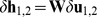 to the fluctuations on the input side are small. This explains why fluctuations of 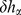 are small in networks stabilized by negative feedback. This argument also shows why the suppression of input-correlations does not rely on a balance between excitation and inhibition; it is as well observed in purely inhibitory networks of leaky integrate-and-fire neurons [17, cf. text following eq. (21) therein] and of binary neurons [52, eq. (30)], where the overall negative feedback suppresses population fluctuations 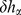 in exactly the same manner, as the only appearing eigenvalue in this case is negative. Figure 5 shows the correlations in a purely inhibitory network without any external fluctuating drive. In this network the neurons are autonomously active due to a negative threshold 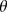, which, by the cancellation argument 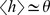, was chosen to obtain a mean activity of about 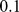. Pairwise correlations in the finite-sized network follow from (23) to be negative,
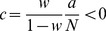
(32)and approach 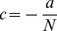 in the limit of strong coupling, as also shown in [52, eq. 30]. The contributions to the input correlation follow from (25) and (26) as
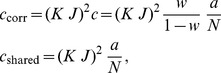
(33)so that for strong negative feedback 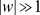 the contribution due to correlations approaches 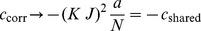. In this limit the two contributions cancel each other as in the inhibition-dominated network with excitation and inhibition. Note, however, that the presence of externally imposed fluctuations is not required for the mechanism of cancellation by negative feedback. The negative feedback suppresses also purely network generated fluctuations. For finite coupling we have 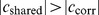, so the total currents are always positively correlated.

**Figure 6:**
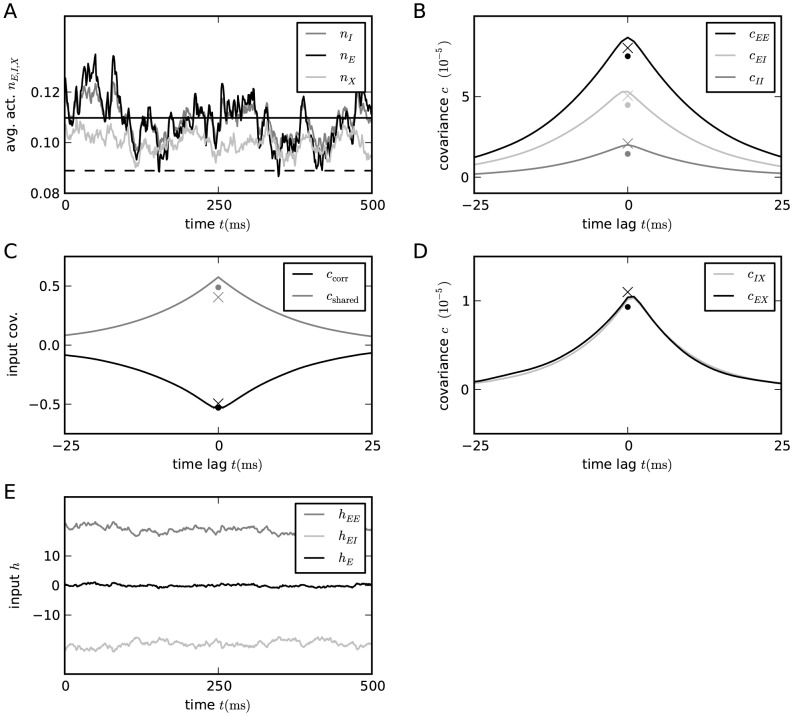
Activity in a network of 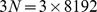 binary neurons with synaptic amplitudes 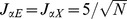, 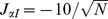 depending exclusively on the type of the sending neuron (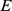 or 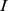). Each neuron receives 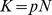 randomly drawn inputs (fixed in-degree, 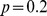). **A** Population averaged activity (black 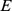, gray 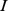, light gray 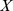). Analytical prediction (5) for the mean activities 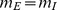 (dashed horizontal line) and numerical solution of mean field equation (7) (solid horizontal line). **B** Cross covariance between excitatory neurons (black), between inhibitory neurons (gray), and between excitatory and inhibitory neurons (light gray). Theoretical results (24) shown as dots. St. Andrew's Crosses indicate the theoretical prediction of leading order in 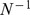 (43). **C** Correlation between the input currents to a pair of excitatory neurons. The black curve is the contribution due to pairwise correlations 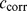, the gray curve is the contribution of shared input 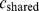. The symbols show the theoretical expectation (25) and (26) based on (43) (crosses) and based on (24) (dots). **D** Similar to B, but showing the correlations between external neurons and neurons in the excitatory and inhibitory population. Note that both theories yield 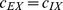, so for each theory ((43) crosses, (24) dots) only the symbol for 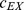 is visible. **E** Contributions 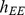 (gray) due to excitatory synapses and 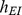 (light gray) due to inhibitory synapses to the input 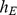 averaged over all excitatory neurons. Duration of simulation 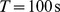, mean activity 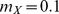, 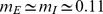, other parameters as in Figure 3.

An interesting special case is a network with homogeneous connectivity, as studied in **“Correlations are driven by intrinsic and external fluctuations”**, where 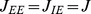 and 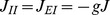, shown in Figure 6. In this symmetric case there is only one negative eigenvalue 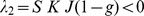. The other eigenvalue is 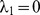, so fluctuations are only mildly suppressed in direction 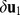. However, on the input side of the neurons, these fluctuations are not seen, since their contribution to the input field is by the vanishing eigenvalue 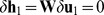. Another consequence of the vanishing eigenvalue is that the system can freely fluctuate along the eigendirection 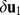. Consequently the tracking of the external signal is much weaker in this case, as evidenced in Figure 6A.

It is easy to see that the cancellation condition (27) does not uniquely determine the structure of correlations in an 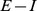 network, i.e. the structure of correlations in a finite network is not uniquely determined by 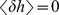. This is shown in Figure 4B, illustrating as an example the correlation structure predicted in the limit of infinite network size and perfect tracking [24, supplement, eqs. 38–39], which fulfills 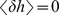 exactly, because this correlation structure can alternatively be derived starting from the condition for perfect tracking 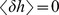. The predicted structure does not coincide with the results obtained by direct simulation of the finite network. By construction and by virtue of (27) this correlation structure, however, still fulfills the cancellation condition on the input side, as visualized in Figure 4C. We show in **“Limit of infinite network size”** below that the deviations from direct simulation are due to the theory being strictly valid only in the limit of infinite network size, neglecting the contribution of fluctuations of the local populations (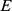,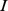), as they appear in (24). Formally this is apparent from [24, eq. (2)] and [24, supplement eq. (40–41)], stating that the solution for correlations is equivalent to the network fluctuations predominantly caused by the external input, also reflected in the expression 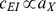 [24, supplement eq. (38–39)]. This can be demonstrated explicitly by setting 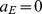 and 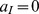 in (24), resulting in a similar prediction for 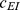, as shown in Figure 4B (plus symbol). The remaining deviation between the theories is due to the different susceptibilities 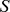 used by the two approaches. The full theory (24) predicts the correct correlation structure independent of the connectivity matrix. In summary, the cancellation condition imposes a constraint on the structure of correlations but is not sufficient as a unique determinant.

The distribution of the in-degree in Figure 4 is an additional source of variability compared to the case of fixed in-degree. It causes a distribution of the mean activity of the neurons in the network, as shown in Figure 4F. The shape of the distribution can be assessed analytically by self-consistently solving a system of equations for the first 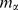 (18) and second moment 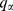 (19) of the rate distribution [54], as described in **“Influence of inhomogeneity of in-degrees”**. The resulting second moments 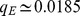 (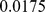 by simulation) and 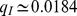 (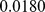 by simulation) are small compared to the mean activity 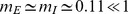. For the prediction of the covariances shown in Figure 4B–D we employed the semi-analytical self-consistent solution to determine the variances 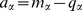. The difference to the approximate value 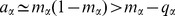 is, however, small for low mean activity.

### Limit of infinite network size

To relate the finite-size correlations presented in the previous sections to earlier studies on the dominant contribution to correlations in the limit of infinitely large networks [24], we here take the limit 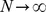. For non-homogeneous connectivity, we recover the earlier result [24] in **“Inhomogeneous connectivity”**. In **“Homogeneous connectivity”** we show that the correlations converge to a different limit than what would be expected from the idea of fast tracking.

Starting from (10) we follow [24, supplement] and introduce the covariances between population-averaged activities as 
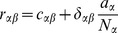
, which leads to
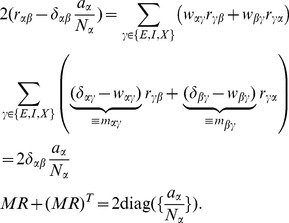
(34)The general solution of the continuous Lyapunov equation stated in the last line can be obtained by projecting onto the set of left-sided eigenvectors of 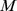 (see e.g. [35] eq. 6.14). Alternatively the system of linear equations (34) may be written explicitly as
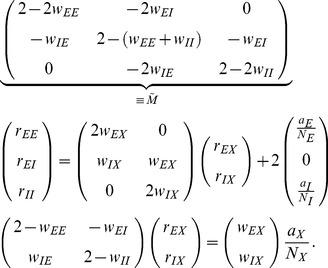
(35)The solution of the latter equation is given by (12), so 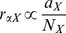. We observe that the right hand side of the first line in (35) contains again two source terms, those corresponding to fluctuations caused by the external drive (proportional to 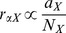) and those due to fluctuations generated within the network (proportional to 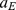 or 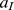). This motivates our definition of the two contributions 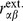 and 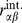 as
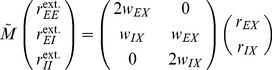
(36)
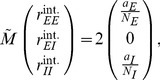
(37)which allows us to write the full solution of (35) as 
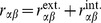
. We use the superscripts 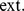 and 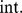 to distinguish the driving sources of the fluctuations coming from outside the network (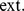 driven by 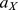) and coming from within the network (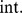 driven by 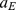 and 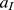).

#### Inhomogeneous connectivity

**Figure 7:**
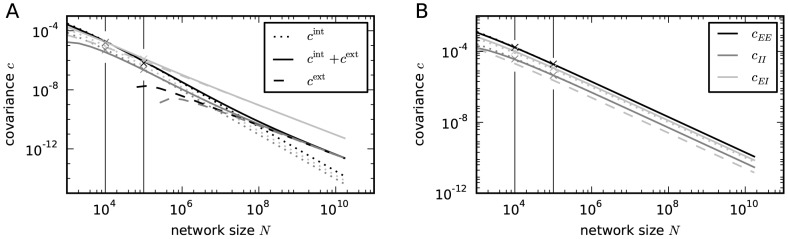
Scaling the network size to infinity. Comparison of the solution of (24) (solid) to the contribution of the leading order in 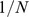 (dashed). Gray coded are the different pairs of covariances, black (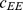), mid gray (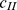), light gray (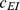). **A** Network as in [24] with non-homogeneous synaptic coupling as in Figure 4. The dashed curve is given by the leading order term 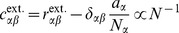 (38) and [24, eqs. (38)–(39)] driven by external fluctuations, the dotted curve is the next order term 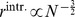 (37), driven by intrinsic fluctuations generated by the excitatory and inhibitory population. The dashed curve is not shown for networks smaller than 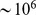 neurons as it assumes negative values. Relative error of the theory with respect to simulation at 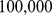 neurons is 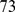 percent. The solid curve is the full solution of (24) 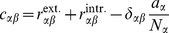. The relative error at 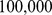 neurons is 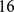 percent. Symbols show direct simulations. **B** Network with homogeneous connectivity, as in Figure 6. Same symbol code as in A. Both contributions 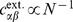 (36) and 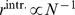 (37) show the same scaling (44). Note that for the parameters here 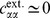, so the only dashed curve shown is 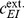. Symbols indicate the results of direct simulations; vertical lines are included to guide the eye.

In the following we assume inhomogeneous connectivity, meaning that the synaptic amplitudes not only depend on the type of the sending neuron but also on the receiving neuron, such that the matrix 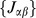 is invertible. In the limit of large networks with 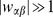 the solution (12) can be approximated as
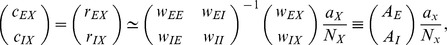
where the definitions of 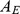 and 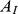 correspond to the ones of [24] if the susceptibility 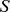 is the same for all populations. Solving the first system of equations (36) leads to
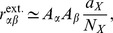
where we again assumed that 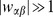 and therefore neglected the 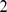 in the sums on the diagonal of the matrix 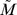 (35). Hence the covariance due to 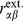 is
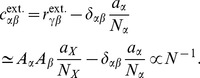
(38)The latter equation is the solution given in [24, supplement, eqs. (38)–(39)]. The form of the equation shows that this contribution is due to fluctuations of the population activity driven by the external input, exhibited by the factor 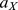 driving 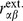, where the intrinsic contribution of the single cell autocorrelations is subtracted. The quantities 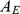 and 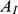 contain the effect of the recurrence on these externally applied fluctuations and are independent of network size, so 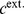 decays with 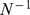 as shown in Figure 7A (dashed curve).

The second contribution 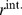 given by the solution of (37) is driven by the intrinsically generated fluctuations. As the network tends to infinity, this contribution vanishes faster than 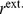, because the coupling matrix grows as 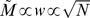. So the term 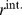 is a correction to (38) of the order 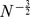. This faster decay can be observed at large network sizes in Figure 7A (dotted curve). For finite networks of natural size, however, this term determines the structure of the correlations. Specifically, for the parameters chosen in [24], the contribution 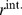 dominates in networks up to about 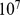 neurons (Figure 7A).

#### Homogeneous connectivity

In the previous section we showed that in agreement with [24] the leading order term 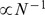 dominates the limit of infinitely large networks and yields practically useful results for random networks of 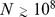 neurons. In the following we will extend the theory to homogeneous connectivity, where the synaptic weights only depend on the type of the sending neuron, i.e. all 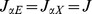 and 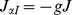 are the same for all 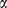. The matrix
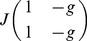
(39)is hence not invertible and the theory in **“Inhomogeneous connectivity”** not directly applicable. Note that assuming fast tracking in this situation, which for inhomogeneous connectivity is a consequence of the correlation structure in the 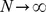 limit [24, eq. (2)], due to the degenerate rows of the connectivity here yields
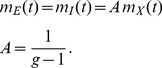
(40)Here the assumption leads to a wrong result, if 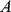 is naively inserted into equation (38) or equivalently into [24, supplement, eqs. (38)–(39)]. In particular, for the given parameters 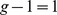 and with homogeneous activity (and 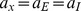) the cross covariances 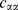 are predicted to approximately vanish 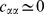. This failure could have been anticipated based on the observation that the tracking does not hold in this case, as observed in Figure 6A. We therefore need to extend the theory for the 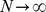 limit of networks with homogeneous connectivity.

To this end we write out (24) explicitly for the homogeneous network using 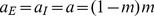. In (24) we observe that 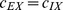 and 
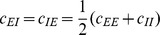
 and introduce 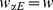, 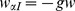, 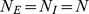 to obtain
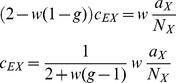
(41)
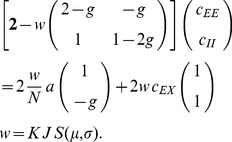
(42)For sufficiently large networks, we can neglect a 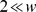 on the left hand side of (41) to obtain
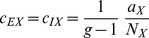
and hence the second equation, again neglecting the 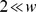 on the left hand side, leads to
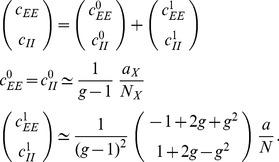
(43)This result shows explicitly the two contributions to the correlations due to external fluctuations (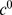) and due to intrinsic fluctuations (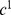), respectively. In contrast to the case of inhomogeneous connectivity, both contributions decay as 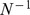, so the external drive does not provide the leading contribution even in the limit 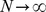. Note also that we may write this result in a similar form as for the inhomogeneous connectivity, as
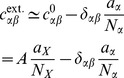
(44)
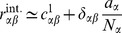

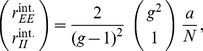
with 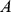 given by (40). Here, 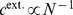 has the same form as the solution [24, eqs. (38)–(39)] originating from external fluctuations, but 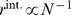 is still a contribution of same order of magnitude. The susceptibility 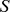 has been eliminated from these expressions and hence only structural parameters remain, analogous to the solution [24, eqs. (38)–(39)]. The two contributions 
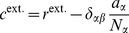
 and 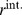 given by the non-approximate solution of (36) and (37), respectively, are shown together with their sum and with results of direct simulations in Figure 7B. For the given network parameters, the contribution of intrinsic correlations dominates across all network sizes, because 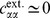, as 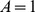, and all 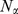 and 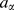 are approximately identical for 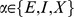. The splitting between the covariances of different types scales proportional to the absolute value 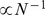, so even at infinite network size the relative differences between the covariances stay the same.

The underlying reason for the qualitatively different scaling of the intrinsically generated correlations 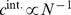 for homogeneous connectivity compared to 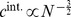 for inhomogeneous connectivity is related to the vanishing eigenvalue of the effective connectivity matrix (39). The zero eigenvalue belongs to the eigenvector 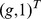, meaning excitation and inhibition may freely fluctuate in this eigendirection without sensing any negative feedback through the connectivity, as reflected in the last line in (44). These fluctuations are driven by the intrinsically generated noise of the stochastic update process and hence contribute notably to the correlations in the network.

In summary, the two examples **“Inhomogeneous connectivity”** and **“Homogeneous connectivity”** are both inhibition-dominated (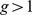) networks that exhibit small correlations on the order 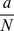 at finite size 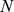. Only in the limit of infinitely large networks with inhomogeneous connectivity is 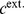 the dominant contribution that can be related to fast and perfect tracking of the external drive. At finite network sizes, the contribution 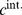 is generally not negligible and may be dominant. Therefore fast tracking cannot be the explanation of small correlations in these networks. Note that there is a difference in the line of argument used in the main text of [24] and its mathematical supplement: While the main text advocates fast tracking as the underlying mechanism explaining small correlations, in the mathematical supplement fast tracking is found as a consequence of the theory of correlations in the limit of infinite network size and under the stated prerequisites, in line with the calculation presented above.

### Influence of connectivity on the correlation structure

**Figure 8:**
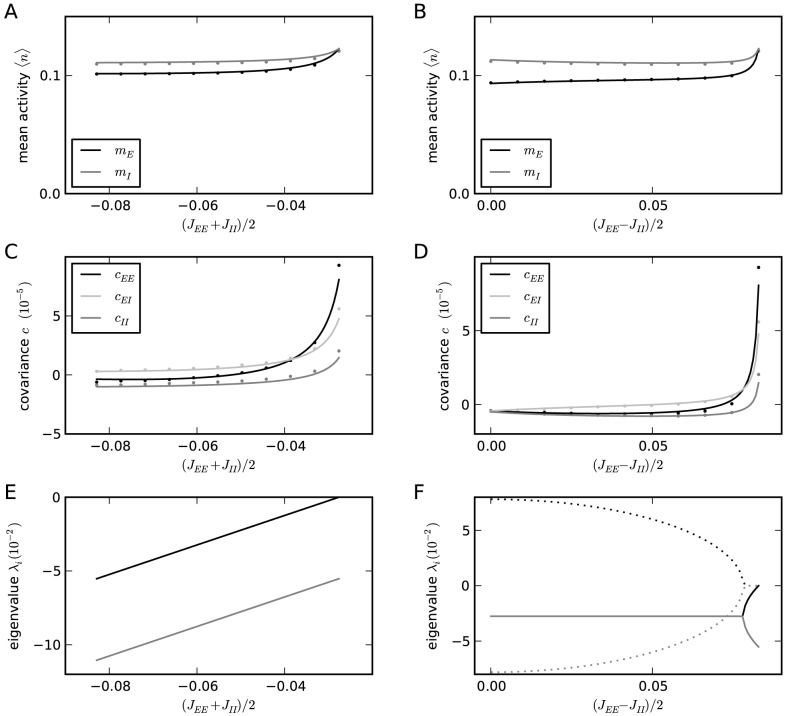
Connectivity structure determines correlation structure. In the left column (A,C,E) 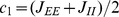 is the independent variable, in the right column (B,D,F) 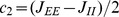. **A**,**B** Mean activity in the network as a function of the structural parameters 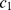 and 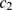, respectively. **C**,**D** Correlations averaged over pairs of neurons. Dots obtained from direct simulation, solid curves given by theory (24) **E**,**F** Eigenvalues (30) of the population-averaged connectivity matrix; solid curves show the real part, dashed curves the imaginary part.

Comparing Figure 6B and Figure 4B, the structure of correlations is obviously different. In Figure 6B, the structure is 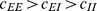, whereas in Figure 4B the relation is 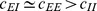. The only difference between these two networks is in the coupling strengths 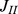 and 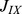. In the following we derive a more complete picture of the determinants of the correlation structure. In order to identify the parameters that influence the fluctuations in these networks, it is instructive to study the mean-field equation for the population-averaged activities. Linearizing (20) for small deviations 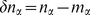 of the population-averaged activity 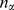 from the fixed point 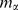, for large networks with 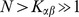 the dominant term is proportional to the change of the mean 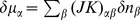, because the standard deviation 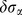 is only proportional to 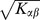. To linear order we hence have a coupled set of two differential equations (29). The dynamics of this coupled set of linear differential equations is determined by the two eigenvalues of the effective connectivity (30). Due to the presence of the leak term on the left hand side of (29), the fixed point rate is stable only if the real parts of the eigenvalues 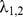 are both smaller than 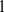. In the network with identical input statistics for all neurons the fluctuating input is characterized by the same mean and variance 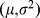 for each neuron. For homogeneous neuron parameters the susceptibility 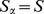 is hence the same for both populations 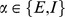. If further the number of synaptic afferents is the same 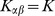 for all populations, the eigenvalues can be expressed by those of the original connectivity matrix as (31)
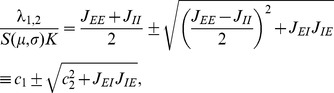
where we defined the two parameters 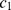 and 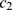 which control the location of the eigenvalues. In the left column of Figure 8 we keep 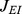, 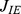, and 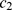 constant and vary 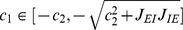, where we choose the maximum value by the condition 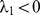 and the minimum value by the condition that 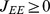 and 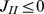, leading to 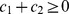 and 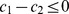, both fulfilled if 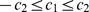. Varying 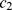 in the right column of Figure 8, the bounds are given by the same condition that 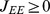 and 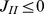, so 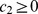, and the condition for the larger eigenvalue to stay below or equal 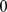, so 
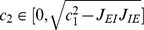
. In order for the network to maintain similar mean activity, we choose the threshold of the neurons such that the cancellation condition 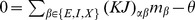 is fulfilled for 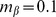. The resulting average activity is close to this desired value of 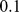 and agrees well to the analytical prediction (20), as shown in Figure 8 A, B.

The right-most point in both columns of Figure 8 where one eigenvalue vanishes 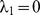, results in the same connectivity structure. This is the case for the connectivity with the symmetry 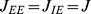 and 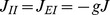 (cf. Figure 6), because in this case the population averaged connectivity matrix has two linearly dependent rows, hence a vanishing determinant and thus an eigenvalue 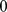. As observed in Figure 8C,D at this point the absolute magnitude of correlations is largest. This is intuitively clear as the network has a degree of freedom in the direction of the eigenvector 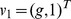 belonging to the vanishing eigenvalue 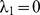. In this direction the system effectively does not feel any negative feedback, so the evolution is as if the connectivity would be absent. Fluctuations in this direction are large and are only damped by the exponential relaxation of the neuronal dynamics, given by the left hand side of (29). The time constant of these fluctuations is then solely determined by the time constant of the single neurons, as seen in Figure 6B. From the coefficients of the eigenvector we can further conclude that the fluctuations of the excitatory population are stronger by a factor 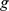 than those of the inhibitory population, explaining why 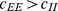, and that both populations fluctuate in-phase, so 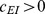, (Figure 8C,D, right most point). Moving away from this point, panels C,D in Figure 8 both show that the magnitude of correlations decreases. Comparing the temporal structures of Figure 6B and Figure 4B shows that also the time scale of fluctuations decreases. The two structural parameters 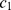 and 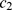 affect the eigenvalues of the connectivity in a distinct manner. Changing 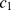 merely shifts the real part of both eigenvalues, but leaves their relative distance constant, as seen in Figure 8E. For smaller values of 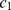 the coupling among excitatory neurons becomes weaker, so their correlations are reduced. At the left most point in Figure 8C the coupling within the excitatory population vanishes, 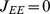. Changing the parameter 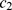 has a qualitatively different effect on the eigenvalues, as seen in Figure 8F. At 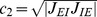, the two real eigenvalues merge and for smaller 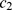 they turn into a conjugate complex pair. At the left-most point 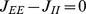, so both couplings within the populations vanish 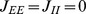. The system then only has coupling from 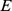 to 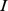 and vice versa. The conjugate complex eigenvalues show that the population activity of the system has oscillatory solutions. This is also called the PING (pyramidal - inhibitory - gamma) mechanism of oscillations in the gamma-range [64]. Panels C,D in Figure 8 show that for most connectivity structures the correlation structure is 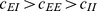, in contrast to our previous finding [17], where we studied only the symmetric case (the right-most point), at which the correlation structure is 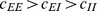. The comparison of the direct simulation to the theoretical prediction (24) in Figure 8C,D shows that the theory yields an accurate prediction of the correlation structure for all connectivity structures considered here.

## Discussion

The present work explains the observed pairwise correlations in a homogeneous random network of excitatory and inhibitory binary model neurons driven by an external population of finite size.

On the methodological side the work is similar to the approach taken in the work of Renart et al. [24], that starts from the microscopic Glauber dynamics of binary networks with dense and strong synaptic coupling 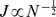 and derives a set of self-consistent equations for the second moment of the fluctuations in the network. As in the earlier work [24], we take into account the fluctuations due to the balanced synaptic noise in the linearization of the neuronal response [24], [65] rather than relying on noise intrinsic to each neuron, as in the work by Ginzburg and Sompolinsky [35]. Although the theory by Ginzburg and Sompolinsky [35] was explicitly derived for binary networks that are densely, but weakly coupled, i.e. the number of synapses per neuron is 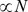 and synaptic amplitudes scale as 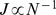, identical equations result for the case of strong coupling, where the synaptic amplitudes decay slower than 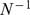 [24]. The reason for both weakly and strongly coupled networks to be describable by the same equations lies in the self-regulating property of binary neurons: Their susceptibility (called 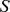 in the present work) inversely scales with the fluctuations in the input, 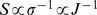, such that 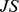 and hence correlations are independent of the synaptic amplitude 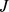 [65]. A difference between the work of Ginzburg and Sompolinsky [35] and the work of Renart et al. [24] is, however, that the former authors assume all correlations to be equally small 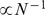, whereas the latter show that the distribution of correlations is wider than their mean due to the variability in the connectivity, in particular the varying number of common inputs. The theory yields the dominant contribution to the mean value of this distribution scaling as 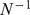 in the limit of infinite network size. Although the asynchronous state of densely coupled networks has been described earlier [42], [54] by a mean-field theory neglecting correlations, the main achievement of the work by Renart et al. [24] must be seen as demonstrating that the formal structure of the theory of correlations indeed admits a solution with low correlations of order 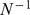 and that such a solution is accompanied by the cancellation of correlations between the inputs to pairs of neurons. In particular can this state of small correlations be achieved although the contribution of shared afferents to the input correlations is of order 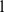 in the strong coupling limit, in contrast to the work of [35], where this contribution is of order 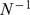. The authors of [24] employ an elegant scaling argument, taking the network size and hence the coupling to infinity, to obtain their results. In contrast, here we study these networks at finite size and obtain a theoretical prediction in good agreement with direct simulations in a large range of biologically relevant networks sizes. We further extend the framework of correlations in binary networks by an iterative procedure taking into account the finite-size fluctuations in the mean-field solution to determine the working point (mean activity) of the network. We find that the iteration converges to predictions for the covariance with higher accuracy than the previous method.

Equipped with these methods we investigate a network driven by correlated input due to shared afferents supplied by an external population. The analytical expressions for the covariances averaged over pairs of neurons show that correlations have two components that linearly superimpose, one caused by intrinsic fluctuations generated within the local network and one caused by fluctuations due to the external population. The size 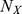 of the external population controls the strength of the correlations in the external input. We find that this external input causes an offset of all pairwise correlations, which decreases with increasing external population size in proportion to the strength of the external correlations (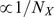). The structure of correlations within the local network, i.e. the differences between correlations for pairs of neurons of different types, is mostly determined by the intrinsically generated fluctuations. These are proportional to the population-averaged variances 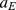 and 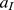 of the activity of the neurons in the local network. As a result, the structure of correlations is mostly independent of the external drive, and hence similar to the limiting case of an infinitely large external population 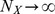 or the case where the external drive is replaced by a DC signal with the same mean. For the other extreme, when the size of the external population equals the number of external afferents, 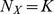, all neurons receive an exactly identical external signal. We show that the mechanism of decorrelation [24], [17] still holds for these strongly correlated external signals. The resulting correlation within the network is much smaller than expected given the amount of common input.

We proceed to re-investigate three observations in balanced random networks: fast tracking of external input signals [42], [54], the suppression of common input correlations, and small pairwise correlations to provide a view that is complementary to previous reports [24], [17], [52]. The lines of argument on these matters provided in the main text of [24] and in its mathematical supplement (as well as in [52]) differ. The main text starts at the observation that in large networks in the inhibition-dominated regime with an invertible connectivity matrix the activity exhibits fast-tracking [24, eq. (2)]. The authors then argue that hence positive correlations between excitatory and inhibitory synaptic currents are responsible for the decorrelation of network activity. The mathematical supplement, however, first derives the leading term of order 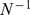 for the pairwise correlations in the network in the limit of infinite network size [24, supplement, eqs. 38,39] and then shows that fast tracking and the cancellation of input correlations are both consequences of this correlation structure. The relation of fast tracking to the structure of correlations is a novel finding in [24, supplement, section 1.4] and not contained in the original report on fast tracking [42], [54]. We here in addition show that the cancellation of correlations between the inputs to pairs of neurons is equivalent to a suppression of fluctuations of the population-averaged input. We further demonstrate how negative feedback suppresses these fluctuations. This argument is in line with the earlier explanation that correlations are suppressed by negative feedback on the population level [17]. Dominant negative feedback is a fundamental requirement for the network to stabilize its activity in the balanced state [42]. We further show that the cancellation of input correlations does not uniquely determine the structure of correlations; different structures of correlations lead to the same cancellation of correlations between the summed inputs. The cancellation of input correlations therefore only constitutes a constraint for the pairwise correlations in the network. This constraint is identically fulfilled if the network shows perfect tracking of external input, which is equivalent to completely vanishing input fluctuations [24]. We show that the correlation structure compatible with perfect tracking [24, supplement, eqs. 38,39] is generally different from the structure in finite-sized networks, although both fulfill the constraint imposed by the cancellation of input correlations.

Performing the limit 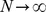 we distinguish two cases. (i) For an invertible connectivity matrix, we recover the result by [24], that in the limit of infinite network size correlations are dominated by tracking of the external signal and intrinsically generated fluctuations can be neglected; the resulting expressions for the correlations within the network [24, supplement, eqs. 38,39] are lacking the locally generated fluctuations that decay faster than 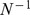 for invertible connectivity. However, the intermediate result [24, supplement, eqs. 31,33] is identical to [35, eq. 6.8] and to (9) and contains both contributions. The convergence of the correlation structure to the limiting theory appears to be slow. For the parameters given in [24], quantitative agreement is achieved at around 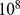 neurons. For the range of network sizes up to which a random network is typically considered a good model (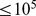 neurons), the correlation structure is dominated by intrinsic fluctuations. (ii) For a singular matrix, as for example resulting from statistically identical inputs to excitatory and inhibitory neurons, the contributions of external and intrinsic fluctuations both scale as 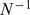. Hence the intrinsic contribution cannot be neglected even in the limit 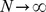. At finite network size the observed structure of correlations generally contains contributions from both intrinsic and external fluctuations, still present in the intermediate result [24, supplement, eqs. 31, 33] and in [35, eq. 6.8] and (9). In particular, the external contribution dominating in infinite networks with invertible connectivity may be negligible at finite network size. We therefore conclude that the mechanism determining the correlation structure in finite networks cannot be deduced from the limit 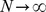 and is not given by fast tracking of the external signal. Fast tracking is rather a consequence of negative feedback.

For the common but special choice of network connectivity where the synaptic weights depend only on the type of the source but not the target neuron, i.e. 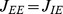 and 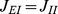 [44], we show that the locally generated fluctuations and correlations are elevated and that the activity only loosely tracks the external input. The resulting correlation structure is 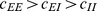. To systematically investigate the dependence of the correlation structure on the network connectivity, it proves useful to parameterize the structure of the network by two measures differentially controlling the location of the eigenvalues of the connectivity matrix. We find that for a wide parameter regime the correlations change quantitatively, but the correlation structure 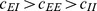 remains invariant. The qualitative comparison with experimental observations of [51] hence only constrains the connectivity to be within the one or the other parameter regime.

The networks we study here are balanced networks in the original sense as introduced in [42], that is to say they are inhibition-dominated and the balance of excitatory and inhibitory currents on the input side to a neuron arises as a dynamic phenomenon due to dominance of negative feedback which stabilizes the mean activity. A network with a balance of excitation and inhibition built into the connectivity of the network on the other hand would correspond in our notation to setting 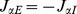 for both receiving populations 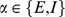, assuming identical sizes for the excitatory and the inhibitory population. The network activity is then no longer stabilized by negative feedback, because the mean activities 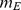 and 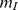 can freely co-fluctuate, 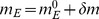 and 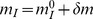, without affecting the input to other cells: 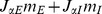 is independent of 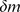. Mathematically this amounts to a two-fold degenerate vanishing eigenvalue of the effective connectivity matrix. The resulting strong fluctuations would have to be treated with different methods than presented here and would lead to strong correlations.

The current work assumes that fluctuations are sufficiently small, restricting the expressions to asynchronous and irregular network states. Technically this assumption enters in form of two approximations: First, the summed input to a cell is replaced by a Gaussian fluctuating variable, valid only if pairwise correlations are weak. Second, the effect of a single synapse on the outgoing activity of a neuron is approximated to linear order allowing us to close the hierarchy of moments, as described in [55]. Throughout this work we show in addition to the obtained approximate solutions the results of simulations of the full, non-linear system. Deviations from direct simulations are stronger at lower mean activity, when the synaptic input fluctuates in the non-linear part of the effective transfer function. The best agreement of theory and simulation is hence obtained for a mean population activity close to 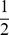, where 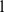 means all neurons are active.

For simplicity in the major parts of this work we consider networks where neurons have a fixed in-degree. In large homogeneous random networks this is often a good approximation, because the mean number of connections is 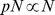, and its standard deviation 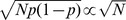 declines relative to the mean. Taking into account distributed synapse numbers and the resulting distribution of the mean activity in Figure 4 and Figure 7A shows that the results are only marginally affected for low mean activity. The impact of the activity distribution on the correlation structure is more pronounced at higher mean activity, where the second moment of the activity distribution has a notable effect on the population-averaged variance.

The presented work is closely related to our previous work on the correlation structure in spiking neuronal networks [17] and indeed was triggered by the review process of the latter. In [17], we exclusively studied the symmetric connectivity structure, where excitatory and inhibitory neurons receive the same input on average. The results are qualitatively the same as those shown in Figure 6. A difference though is, that the external input in [17] is uncorrelated, whereas here it originates from a common finite population. The cancellation condition for input correlations, also observed in vivo [50], holds for spiking networks as well as for the binary networks studied here. For both models, negative feedback constitutes the essential mechanism underlying the suppression of fluctuations at the population level. This can be explained by a formal relationship between the two models (see [53]).

Our theory presents a step towards an understanding of how correlated neuronal activity in local cortical circuits is shaped by recurrence and inputs from other cortical and thalamic areas. For example the correlation between membrane potentials of pairs of neurons in somatosensory cortex of behaving mice is dominated by low-frequency oscillations during quiet wakefulness. If the animal starts whisking, these correlations significantly decrease, even if the sensory nerve fibers are cut, suggesting an internal change of brain state [5]. Our work suggests that such a dynamic reduction of correlation could come about by modulating the effective negative feedback in the network. A possible neural implementation is the increase of tonic drive to inhibitory interneurons. This hypothesis is in line with the observed faster fluctuations in the whisking state [5]. Further work is needed to verify if such a mechanism yields a quantitative explanation of the experimental observations.

The network where the number of incoming external connections per neuron equals the size of the external population, cf. Figure 3 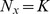, can be regarded as a setting where all neurons receive an identical incoming stimulus. The correlations between this signal and the responses of neurons in the local network (Figure 3C) are smaller than in an unconnected population without local negative feedback. This can formally be seen from (29), because negative eigenvalues of the recurrent coupling dampen the population response of the system. This suppression of correlations between stimulus and local activity hence implies weaker responses of single neurons to the driving signal. Recent experiments have shown that only a sparse subset of around 10 percent of the neurons in S1 of behaving mice responds to a sensory stimulus evoked by the active touch of a whisker with an object [4]. The subset of responding cells is determined by those neurons in which the cell specific combination of activated excitatory and inhibitory conductances drives the membrane potential above threshold. Our work suggests that negative feedback mediated among the layer 2/3 pyramidal cells, e.g. through local interneurons, should effectively reduce their correlated firing. In a biological network the negative feedback arrives with a synaptic delay and effectively reduces the low-frequency content [17]. The response of the local activity is therefore expected to depend on the spectral properties of the stimulus. Intuitively one expects responses to better lock to the stimulus for fast and narrow transients with high-frequency content. Further work is required to investigate this issue in more detail.

A large number of previous studies on the dynamics of local cortical networks focuses on the effect of the local connectivity, but ignores the spatio-temporal structure of external inputs by assuming that neurons in the local network are independently driven by external (often Poissonian) sources. Our study shows that the input correlations of pairs of neurons in the local network are only weakly affected by additional correlations caused by shared external afferents: Even for the extreme case where all neurons in the network receive exactly identical external input (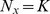), the input correlations are small and only slightly larger than those obtained for the case where neurons receive uncorrelated external input (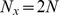; black curve in Figure 8C). One may therefore conclude that the approximation of uncorrelated external input is justified. In general, this may however be a hasty conclusion. Tiny changes in synaptic-input correlations have drastic effects, for example, on the power and reach of extracellular potentials [34]. For the modeling of extracellular potentials, knowledge of the spatio-temporal structure of inputs from remote areas is crucial.

The theory of correlations in presence of externally impinging signals is a required building block to study correlation-sensitive synaptic plasticity [66] in recurrent networks. Understanding the emerging structure of correlations imposed by an external signal is the first step in predicting the connectivity patterns resulting from ongoing synaptic plasticity sensitive to those correlations.
